# J. F. Henkel’s Anleitung Zum Chirurgischen Verbande

**Published:** 1841-05

**Authors:** 


Art. III.—*J. F. Henkel's Anleitung zum Chirurgischen Verbande. Umgearbeitet und mit vielen Zusatzen versehen von Dr. J. C. Stark. Von neuem bearbeitet und mit Zusatzen vermehrt von J. F. Dieffenbach, M. D.*

An Introduction to the Methodic Application of Bandages. By J. F. Henkel, M. D. With forty copper-plates. 12mo. pp. 540. Vienna: 1840.

The first edition of this work was published in 1756, under the title of "Instructions for the Methodic Application of Surgical Bandages," in a small duodecimo volume, and has ever since been the standard authority for reference with German students. The fifth edition, greatly improved and enlarged, was issued in 1790, a short time before the author's death. It reappeared in the early part of the present century with emendations and copious additions from the pen of Dr. Stark; and the edition now before us was published a year ago by Dr. J. F. Dieffenbach, of Berlin, well known as one of the most distinguished surgeons of the age. The text, which is closely printed, embraces five hundred and forty duodecimo pages, and is illustrated by forty plates, executed on copper in the best style of the art. That this work should have sustained itself for nearly a century in the estimation of our German brethren, is not only a striking evidence of its intrinsic value, but an anomaly in the history of medical authorship. This is the more surprising when it is remembered that the amount of competition in this department has by no means been slight; for, besides the essays of Kühn, Hofer, Zauner, Bernstein, Theden, Böttcherr, and Köhler, which all appeared within a very short time of each other, towards the close of the last century, the elaborate manuals of Schreger of Erlangen, and Benedict of Leipsic, which were issued, the one in 1820 and the other in 1827, have had an extensive circulation, and are justly regarded as among the most valuable contributions to surgical science within the last twenty years. About the same period also that the latter work appeared, a translation of the celebrated production of M. P. R. Gerdy of Paris—"Traite des Bandages et Appareils de Pensemens"—was published at Weimar, with copious notes by the German editor. Thus it is evident, as was before intimated, that the treatise of Henkel could have maintained its character as a standard authority, during the long period above mentioned, solely by its intrinsic excellence, and not because of an extraordinary concurrence of circumstances; though doubtless not a little of the reputation which it at present enjoys is to be ascribed to the valuable additions of its two editors, Stark and Dieffenbach.

As it now presents itself, Henkel's "Introduction" affords a complete digest of the existing state of the art of bandaging, and may be considered as the most able production of the kind in any language. It is far superior to the little treatise of Mr. Cutter, and should be in the library of every intelligent surgeon. It is worthy of being well translated, and would doubtless be highly acceptable to the practitioners of this country, to whom we can safely recommend it as a useful and satisfactory guide.

